# The Type of Per-Cooling Strategies Currently Employed by Competitive and Professional Cyclists-Triathletes During Training and Competition Are Condition (Dry vs. Humid) Dependant

**DOI:** 10.3389/fspor.2022.845427

**Published:** 2022-05-25

**Authors:** Freya Bayne, Sebastien Racinais, Katya N. Mileva, Steve Hunter, Nadia Gaoua

**Affiliations:** ^1^Sport and Exercise Science Research Centre, School of Applied Sciences, London South Bank University, London, United Kingdom; ^2^Research and Scientific Support Department, Aspetar, Doha, Qatar

**Keywords:** environmental, temperature, humidity, cooling strategies, cycling performance

## Abstract

**Purpose:**

To investigate cooling strategies employed by athletes (cyclists-triathletes) during training and competition in hot and dry (HD) and hot and humid (HH) conditions.

**Methods:**

Thirty-five athletes completed an online questionnaire on the type, timing, and justification of cooling strategies employed during past training and/or competitions in HD and HH conditions. In addition, 3 athletes also completed a one-to-one follow-up interview.

**Results:**

Comparisons between strategies employed in all conditions were based on *N* = 14 (40%). Cold-water pouring was the most employed (*N* = 4; 21%) strategy during training and/or competing in hot conditions. The timing of the strategies employed was based on pitstops only (*N* = 7; 50%). The justification for strategies employed was based on trial and error (*N* = 9, 42.85%: *N* = 10, 47.61%). All athletes rated strategies employed as 1 (“not effective for minimising performance impairments and heat-related illnesses”). Comparisons between HD and HH were based on *N* = 21 (60%), who employed different strategies based on condition. Cold-water ingestion was the most employed (*N* = 9, 43%) strategy in HD, whereas a combination of cold-water ingestion and pouring was the most employed (*N* = 9, 43%) strategy in HH. The timing of strategies employed in the HD split was pre-planned by distance but was modified based on how athletes felt during (*N* = 8, 38%), and pre-planned by distance and pit stops (*N* = 8, 38%). The timing of strategies employed in HH was pre-planned based on distance and how athletes felt during (*N* = 9, 42%). About 57% (*N* = 12) of the 60% (*N* = 21) perceived effectiveness in HD and HH as 3 (“*Sometimes effective and sometimes not effective”*), whereas 43% (*N* = 9) of the 60% (*N* = 21) perceived effectiveness in HD and HH as 4 (“*Effective for minimising performance impairments*”).

**Conclusion:**

Cold-water ingestion is the preferred strategy by athletes in HD compared to a combination of cold-water ingestion and pouring in HH conditions. All strategies were pre-planned and trialled based on distance and how athletes felt during training and/or competition. These strategies were perceived as effective for minimising performance impairments, but not heat-related illnesses. Future studies should evaluate the effectiveness of these cooling strategies on performance and thermoregulatory responses in HD and HH conditions.

## Introduction

When humidity is low and skin temperature (T_sk_) is high, sweat secreted onto bare skin is readily evaporated and heat can be transferred at high rates from the body to the environment (Maughan et al., 2012). However, when the humidity (interdependent on ambient temperature) of the environment is high, the rate at which sweat evaporates from the skin is lower than in hot and dry conditions (HD; Maughan et al., 2012). The human ability to cool is ~13–17% greater in HD compared to hot and humid conditions (HH) at an equivalent wet-bulb globe temperature (WBGT) value of 32.38°C and activity velocity of 0.3–0.7 ms^1^ (Vanos and Grundstein, 2020). The impairment in sweat rate (SR) in HH results in an imbalance between heat loss and heat gain, leading to an increase in core/rectal temperature (T_core_/T_rectal_) and T_sk_ (Maughan et al., 2012; Teunissen et al., 2013). These physiological responses are commonly accompanied by psychological responses, such as a progressive decline in thermal comfort (TC; from comfortable to uncomfortable; Moss et al., 2021), and an increase in ratings of perceived exertion (RPE; from light to maximal effort; Moss et al., 2021), and thermal sensation (TS; from cool to hot; Moss et al., 2021). These perceptual responses differ between individuals and environmental conditions (HD vs. HH; Gaoua, 2010). In terms of capacity and performance, HH has a greater detrimental effect on exercise capacity (Maughan et al., 2012) and time-trial performance (Teunissen et al., 2013) compared to HD. However, more recent findings by Lei et al. (2020), where conditions were matched for vapour pressure/absolute humidity contradict Teunissen et al. (2013) findings, reporting no difference in time-trial performance between HH and HD. Due to the scarcity of research comparing HD and HH, the exact effect of combined heat and humidity on performance is unclear. In addition to performance impairments, athletes may suffer from heat-related illnesses (HRI) whilst training and/or competition in hot conditions, such as heat exhaustion, exertional heat injury, and exertional heat stroke (Winkenwerder and Sawka, 2007).

To minimise performance impairments and HRI, athletes incorporate heat mitigation strategies, such as heat acclimation/acclimatisation, cooling, and/or hydration before or during training and/or competition (Gibson et al., 2020). Heat acclimation/acclimatisation is a chronic heat mitigation strategy in which the aim is to induce heat adaption and enhance an individual's ability to tolerate heat stress (Gibson et al., 2020). The adaptation from this strategy can be maintained for ~8weeks with intermittent heat training once a week (Sekiguchi et al., 2021). However, no intermittent heat training following heat acclimation/acclimatisation will result in a loss of adaptations after 4 weeks and even greater losses after 8 weeks (Sekiguchi et al., 2021). Due to the length, nature, and cost of this strategy, some athletes opt for acute heat mitigation strategies, such as cooling and/or hydration that can be applied easily and cost-effectively during training and/or competition (Gibson et al., 2020). In HH, cold-water ingestion provides mechanistic benefits, whereas, in HD, cold-water pouring/dousing provides mechanistic benefits (Morris and Jay, 2016). An investigation of the hydration and cooling strategies employed during the Doha 2019 IAAF World Athletics Championships which was in HD, revealed that 93% of endurance athletes employed a pre-planned drinking strategy including water (85%), electrolytes (83%), and carbohydrates (81%; Racinais et al., 2021). These strategies were based on personal experience rather than evidence-based, which suggests limited empirical research into practise. Moreover, 80% of endurance athletes employed pre-cooling (mainly ice-vest 53% and cold-towel 45%), and 93% employed per-cooling [mainly head/face water dousing/pouring (65%) and cold-water ingestion (52%)], which were widely pre-planned by athletes. Ice-slurry ingestion (11–21%) and menthol-based interventions (1–2%) were less common. The strategies employed by this elite population in HD coincide with Morris and Jay's (2016) recommendations. However, the perceived effectiveness of these strategies during competition in HD was not measured or reported. Perceptual responses such as TC, TS, RPE can offer an insight into the perceived effectiveness of cooling strategies during training and/or competition. For example, a review by, Douzi et al. (2020) stated that per-cooling strategies improved perceptual measures such as thermal perception and RPE, thereby inducing better self-selected intensities during time trials.

The timing of application of heat mitigation strategies, as well as the type of heat mitigation strategy, is vital for athletes' competition preparation in hot conditions such as the recent 2021 Tokyo Summer Olympic Games (Gibson et al., 2020). Lei and Wang (2021) highlighted that the combination of high humidity and ambient temperature of the 2021 Tokyo Summer Olympic Game would undoubtfully result in greater physiological strains and, thereby, downregulate the endurance performance of athletes. Although many research studies have highlighted that the thermoregulatory strain is greater in HH, no review articles have directly compared HD and HH and associated thermoregulatory performance, and such a lack of consensuses in this area will lead to an increase in the risk of HRI and suboptimal preparation. Therefore, Lei and Wang (2021) highlighted the potential interventional strategies to reduce thermal strain in HH including pre-cooling *via* cold-water immersion with proper hydration and some potential per-cooling modalities such as cold-water/ice-slushy ingestion. Following this, Lei and Wang (2021) recommended that these strategies be trialled, as the research in HD and HH is far from complete. Therefore, the purpose of this study was to investigate the type, timing, and justification of per-cooling strategies currently employed by cyclists-triathletes during training and/or competitions in HD and HH.

## Materials and Methods

### Study Population

This study was aimed at cyclists and triathletes who have trained or competed in hot conditions. The questionnaire was sent *via* email to cycle and triathlon clubs and advertised on social media channels, such as Twitter®, Instagram®, and Facebook®. Participation in the questionnaire was open to all genders, all levels of participation, and ages (Table 1). It was advised that participation was not permitted, or data was rejected if the participant had not trained or competed in hot conditions (≥28°C; Table 1).

**Table 1:** Table outlines inclusion and exclusion criteria for participants.

**Inclusion**	**Exclusion**
All sex (M:F) and genders (Transgender, non-binary).	Do not train or compete in hot conditions (≥28°C).
All levels of participation (e.g. recreational, competitive, professional).	
Sporting background: Cycling and/or Triathlon.	Live in countries that are hot (≥28°C) for more than half of the year (acclimatization effect).
All ages.	

The 35 participants were separated into three categories: recreational, competitive, and professional. Recreational athletes were classed as individuals that participate in the sport for enjoyment, fitness, and/or social reasons (*N* = 10; Table 2). Competitive athletes were classed as individuals who competitively participate in and train for competition (*N* = 15; Table 2); whereas. professional athletes held a pro licence and participated in competitions for fiscal rewards and/or representation of their country (*N* = 10; Table 2).

**Table 2:** Participant characteristics including number of participants, sex ratio (M:F), country participants live in, years of experience (yrs), number of competitions competed in, number of training sessions in hot conditions, percentage of group that have experienced symptoms related to heat related illnesses (%).

**Participants**	**No. of participants**	**Sex ratio** **(% of M:F)**	**Age (yrs)**	**Country participants live in**	**Experience (yrs) (*N =* 13)**	**No. Of competitions competed in**	**No. of training sessions or competitions in hot conditions**.	**Percentage of group that have experienced symptoms related to heat related illnesses (%)**
**All**	35	71(30% was T):29	35 ± 12	UK = 89% Germany = 7% Romania = 2% Slovenia = 2%	8 ± 5	18 ± 15	4 ± 2	37%
**Recreational**	10	80 (10% was T):20	43 ± 17	UK = 80% Germany = 10% Romania = 10%	9 ± 6	0	2 ± 1	40%
**Competitive**	15	74:26	33 ± 10	UK = 87% Slovenia = 7% Germany = 7%	8 ± 6	26 ± 15	3 ± 1	53%
**Professional**	10	60:40	30 ± 4	UK = 100%	6 ± 2	28 ± 2	6 ± 4	10%

M, male; F, female; T, transgender; yrs, years; No., Number. ^*^Significant difference between groups.

### Study Design

**Figure 1:**
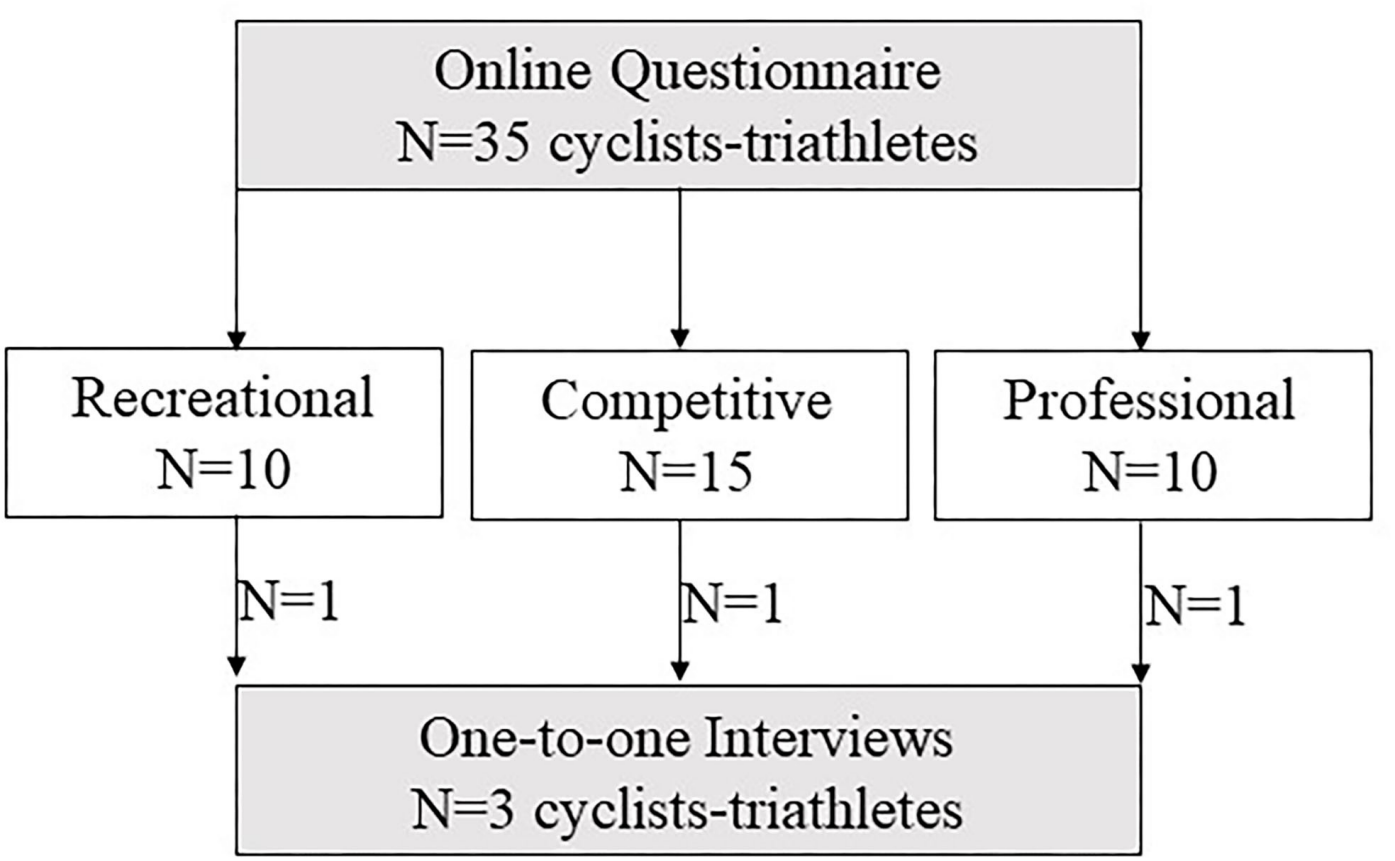
Methodological flow chart of the study design. Grey boxes, data collection; white boxes, participants.

The study had a mixed-methods design. To meet the aim of the current study, 35 participants (Recreational = 10, Competitive = 15, and Professional = 10; Table 2) completed an online questionnaire (Figure 1). To support the questionnaire findings and gain an in-depth understanding of the participant's experience, the investigators “*deliberately sought out individuals or groups who fit the bill”* (Greenhalgh, 1997; p.157). Therefore, convenience sampling was employed to select 3 of the 35 athletes who best represented their sub-groups (1 = Recreational, 1 = Competitive, and 1 = Professional). For the 3 case studies, a follow-up focused interview was used (Figure 1).

### Design of Questionnaire

The questionnaire was created using the online platform https://www.onlinesurveys.ac.uk/. The scope of the questionnaire was to identify heat mitigation strategies type, the timing of application, justification for type and timing, and perceived effectiveness in HD and HH conditions. All questions allowed for text box answers for participants to describe strategies in more detail. The questionnaire was piloted and reviewed by two members of staff at LSBU who were familiar with both cycling exercises and conducting and interpreting questionnaires. It was suggested to adapt questions from a previous questionnaire used in the literature (Racinais et al., 2021) to allow for reflective practise (see examples below). While the focus of the study of Racinais et al. (2021) was to investigate heat mitigation strategies before and during the 2019 Doha World Championships in HD, our study investigated per-cooling strategies (only) during training and/or competition in both HD and HH.

Type: Racinais et al. (2021) asked “what pre-cooling method(s) are you planning on using during the time-trial?” which was adapted to “What per-cooling method(s) did you use during training and/or competition in hot and dry conditions?”

Timing: When was this (these) strategy (ies) applied during hot and dry conditions?

Justification: What was your justification (“why?”) for using this type of strategy and the timing of application in hot and dry conditions?

Perceived effectiveness: Please rate the effectiveness of the strategy employed (type and timing together) in hot and dry conditions on a scale from 1 to 5 (*1* = *Not effective for minimising performance impairments and suffering heat-related illnesses, 2* = *Not effective for minimising performance impairments, 3* = *Sometimes effective and sometimes not effective, 4* = *Effective for minimising performance impairments, 5* = *Effective for minimising both performance impairments and suffering from any heat-related illnesses*). Perceived effectiveness was defined as the athlete's opinion based on their own experiences without an objective measure of performance of heat-related illness.

Participants were also asked to respond to these questions for hot and humid (HH) conditions or hot conditions in general if no difference in condition was perceived at the start of the questionnaire.

### Design of Single Case Study Interviews

A single case study approach was selected to provide an in-depth understanding of the participant's experiences and therefore produce a high-quality theory for future work to expand on. To achieve this, the single case studies were designed and reported in accordance with McKay and Marshall's (2000) checklist and Keegan et al. (2017) guidelines. The single case study consisted of one-to-one interviews that were conducted online *via* zoom (lasting ~30min, record with cameras on). Participants were informed that the interviews were informal, semi-structured, followed a discussion format, and that there were no wrong or right answers.

The interview questions were developed based on answers reported in the questionnaires together with information gathered from a pilot interview conducted with members of staff at LSBU who were familiar with conducting interviews. Therefore, the questions related to the research topic (type, timing, justification, and perceived effectiveness of heat mitigation strategies in HD and HH) are based on the reviewer's feedback. It was also highlighted that the questions needed to be reworded for clarification of different conditions that are related to the interviewee: Therefore, the interviewer gave examples of locations and events that the athletes may have trained or competed in to ensure the interviewee's understanding of environmental conditions. Based on this information the participants selected specific training sessions and/or competitions to discuss for the interview. If known, dates of training and/or competitions were provided by participants, and weather conditions were cross-referenced by two investigators using https://www.metoffice.gov.uk/.

Key terminology was also defined at the start of the questionnaire and interview. For example, cold water was defined as water at 9°C (Gibson et al., 2020) and represented temperature at the start of ingestion and not throughout the exercise entirety.

### Data Analysis

The questionnaire was analysed using the analyse function on https://www.onlinesurveys.ac.uk/, which expressed values as percentages. This was subsequently extracted into Excel. The outcome variables from the questionnaire were:

1. Percentage of participants that employed a specific type of strategy (for example, cold-water ingestion, cold-water pouring, ice packs, ice vests, cooling collars, ice slushy ingestion, menthol, etc.).
2. Percentage of participants that employed a specific time to apply strategies (for example, pre-planned based on distance, pre-planned based on time, pit stops, and how they felt during, etc.).
3. Percentage of participants that had specific justifications for type and timing of strategy (for example, personal reading, sports scientist, previous experience, etc.).
4. Percentage of participants based on ratings of perceived effectiveness (*1* = *Not effective for minimising performance impairments and suffering heat-related illnesses, 2* = *Not effective for minimising performance impairments, 3* = *Sometimes effective and sometimes not effective, 4* = *Effective for minimising performance impairments, and 5* = *Effective for minimising both performance impairments and suffering from any heat-related illnesses*).

The interviews were recorded (video and audio). At the start of the transcription stage, the participants were anonymised through pseudonyms. Transcriptions were imported to NVIVO software for thematic coding. Braun and Clarke's (2006) six-phase analysis process was adopted:

1. Familiarising/immerse yourself with your data: The primary and secondary investigators separately read through the interview transcripts at least 3 times.
2. Generating initial codes: The primary and secondary investigators generated initial codes separately, and then, came together to discuss and determine codes.
3. Searching for themes: The primary and secondary investigators drew out common themes and meanings within each interview separately, and then, came together to discuss and determine themes.
4. Reviewing themes: Common patterns in the data were identified and organised into themes and sub-themes to connect shared experiences in the different interviews.
5. Defining and naming themes: The primary and secondary investigators defined and named themes separately and then came together to discuss and determine definitions and names for themes.
6. Producing the report: These themes were examined to conclude the data, which reflected the different perspectives on training and competing in hot conditions.

## Results

### Type of Per-Cooling Strategy

**Figure 2:**
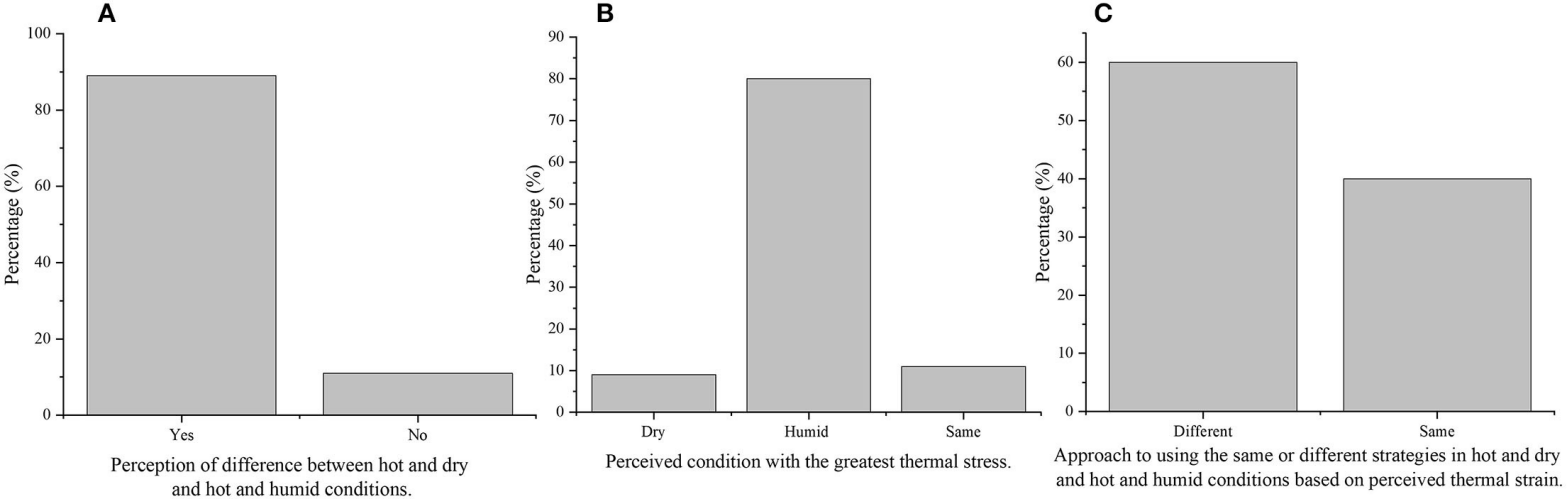
**(A)** Perceived difference between hot and dry and hot and humid conditions, **(B)** perceived condition with the greatest thermal stress, and **(C)** approach to using the same or different strategies in hot and dry and hot and humid conditions based off perceived thermal strain.

Eighty-eight percent (*N* = 31) of participants perceived that there is a difference in thermal stress between HD and HH (Figure 2A). Eighty percent (*N* = 28) of the participants that perceived a difference reported that HH provided greater thermal stress compared to HD (Figure 2B). Only 60% (*N* = 21) of the recruited cohort reported employing different strategies depending on the environmental condition (HD or HH; Figure 2C). Therefore, the results will focus on this 60% (*N* = 21) because the 40% (*N* = 14) that reported employing the same strategies in both HD and HH were due to lack of experience (competitive/recreational level) and were not interested in performance or health outcomes.

Of the 60% (*N* = 21), participants reported seven main strategies employed for cooling during training and/or competition in HD conditions (Table 3).

**Table 3:** Athletes' classification and their most prevalent descriptive key terms.

**Higher order theme**	**Type**	**Timing**	**Justification**	**Perceived effectiveness**
Different HAS used in hot and humid	Cold-water ingestion and pouring Cold-water ingestion and ice-slushy Cold-towels only Cold-water ingestion and pouring, and ice-slushy Cold-water ingestion only Cold-water pouring and cold-towels	Pre-planned by distance and pitstops Pitstops only How they felt during and pitstops Pre-planned by distance and how they felt during performance	Previous experience/perceived effectiveness Personal research Support Staff Previous experience/perceived effectiveness and support staff Cooling availability	57% (*N =* 12) of the 60% (*N =* 21) = 3 (“*Sometimes effective and sometimes not effective”*). 43% (*N =* 9) of the 60% (*N =* 21) = 4 (“*Effective for minimizing performance impairments*”).
Different HAS used in Hot and Dry	Cold-water ingestion and pouring Cold-water towels only Ice vest only Cold-water ingestion and cold-towels Cold-water pouring and cold-towels Cold-water ingestion and pouring, and cold-towels	Pre-planned by distance and how they felt during Pre-planned by distance and pitstops Pre-planned by distance, pitstops and how they felt during performance How they felt during only Pre-planned by distance only	Previous experience/perceived effectiveness Personal research Support Staff Cooling availability No justification/unsure	57% (*N =* 12) of the 60% (*N =* 21) = 3 (“*Sometimes effective and sometimes not effective”*). 43% (*N =* 9) of the 60% (*N =* 21) = 4 (“*Effective for minimizing performance impairments*”).
Same HAS used in all hot conditions	Cold-water pouring only Cold-towels only No per-cooling Cold-water ingestion and pouring Ice-slushy ingestion only Cold-water pouring and cold-towels	Pitstops only How they felt during performance No per-cooling Pre-planned by elapsed time	Previous experience/perceived effectiveness Previous experience/perceived effectiveness and cooling availability No justification/unsure Cooling availability	100% (*N =* 14) = 1 (“not effective for minimizing performance impairments and heat related illnesses).

**Figure 3:**
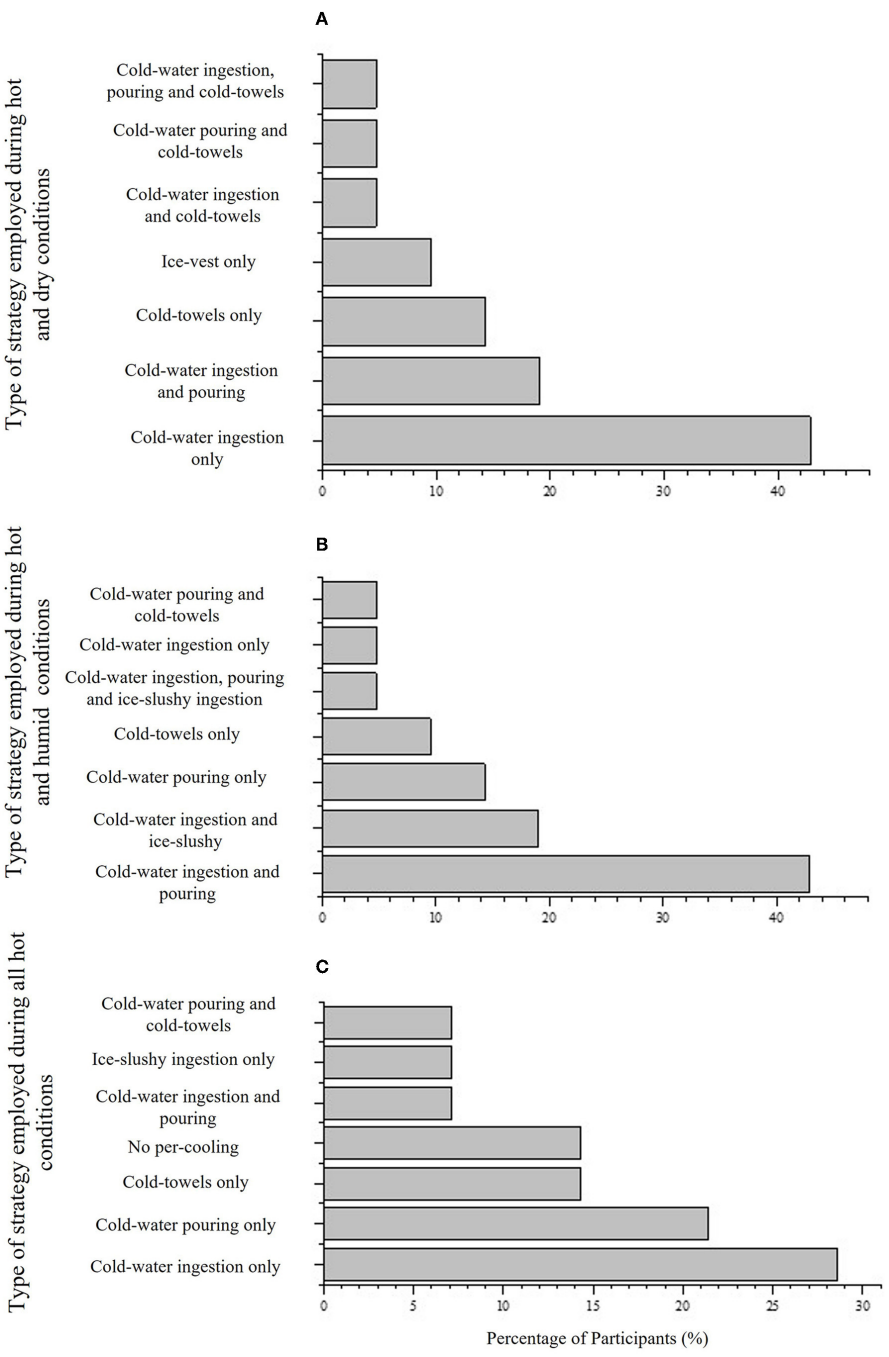
**(A)** Strategies employed in hot and dry conditions by 60% (*N* = 21) participants who use different strategies depending on condition, **(B)** strategies employed in hot and humid conditions by 60% (*N* = 21) participants who use different strategies depending on condition, and **(C)** strategies employed in all hot conditions by 40% (*N* = 14) participants who employed the same strategy regardless of condition.

In HD the prevailing preference was cold-water ingestion [43% (*N* = 9); Figure 3A], followed by cold-water ingestion and pouring [19% (*N* = 4); Figure 3A]; whereas in HH a combination of cold-water ingestion and pouring was the prevailing preference [43% (*N* = 9); Figure 3B], followed by cold-water and ice-slushy ingestion [14% (*N* = 3); Figure 3B].

Some athletes employed different strategies in HD, but others did not, for example, a competitive athlete stated:

“I feel like I ended up using everything possible, I had cold water in coolers at transition, which I would use to drink and pour over myself, and I also had cold towels to apply during the transition.”

Whereas a professional athlete stated:

“For IRONMAN Oman everything was about drinking cold water for me.”

Notably, the strategies employed in HH conditions were different compared to HD for both the competitive and professional athletes. The competitive athlete employed a combination of cold water (ingestion and pouring) and ice slushies:

“I used cold-water ingestion, cold-water pouring, and also ice slushies.”

Whereas the professional athlete employed cold-water only (ingestion and pouring):

“I was drinking cold water again like I did at OMAN but this time I also poured cold water over myself on the bike and the run.” and “I try to target my head, face, neck, and back.”

Mental strategies were also employed amongst the athletes. The competitive athlete reported using imagery and modelling:

“I think because I have gotten used to using the cooling strategies and focusing on implementing them into my races that I actually think about and imagine using them throughout the race so if I know I have a transition coming up where I have an opportunity to use a cooling strategy like a cold towel or cold-water from my cooler then I think about that when I am racing.”

Whereas the professional athlete reported using positive self-talk (Latinjak et al., 2018) and locus of control (Lefcourt, 2014).

“For me, it's about going into a race feeling confident. I remember when I had just started racing and I went to IRONMAN NICE which was HD I think, and I was talking to some of the other competitors before the race and they were talking about other competitions that they had done before whereas I hadn't really done any, but I said to them that I had come to the race to win and that I was going to win […], I make sure that I have controlled everything that I can control prior to the race so that on race day I feel confident that I will win.”

### Timing of Application of Per-Cooling Strategies

**Figure 4:**
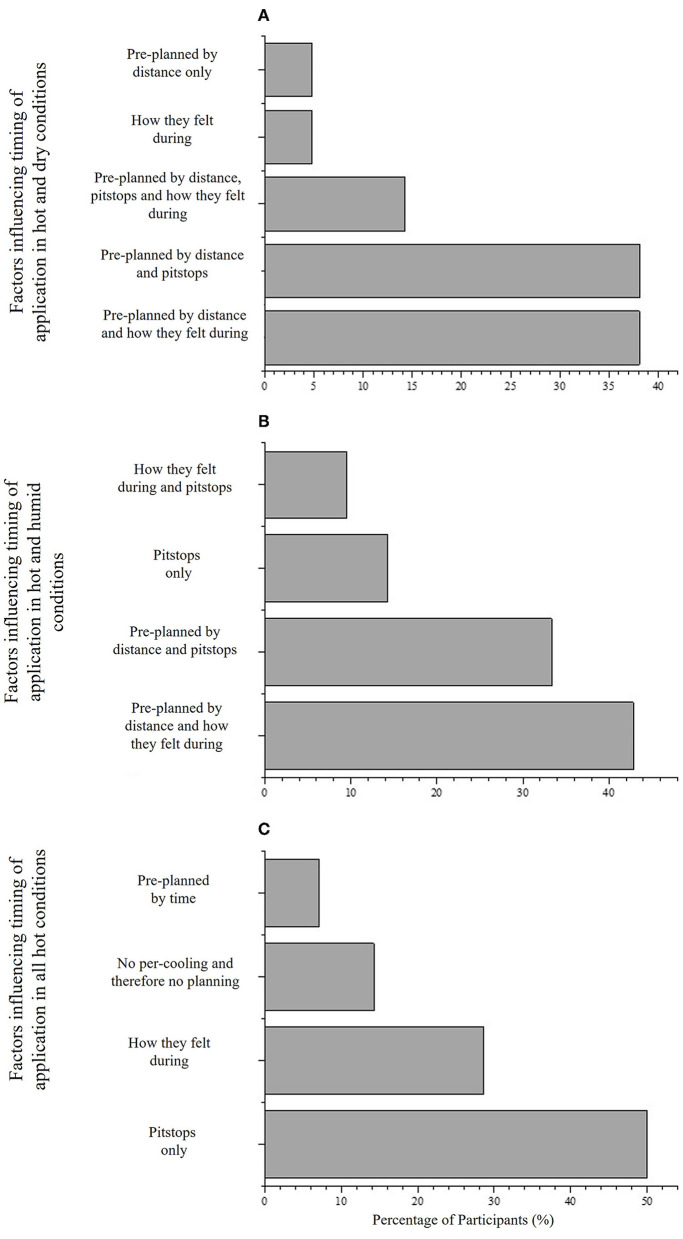
**(A)** Timing of cooling strategies employed in hot and dry conditions by 60% (*N* = 21) participants who use different strategies depending on condition, **(B)** timing of cooling strategies employed in hot and humid conditions by 60% (*N* = 21) participants who use different strategies depending on condition, and **(C)** timing of strategies employed in all hot conditions by 40% (*N* = 14) participants who employed the same strategy regardless of condition.

Participants selected five defining factors that influenced the timing of application in HD (Table 3); whereas only 4 defining factors were reported in HH (Table 3). The prevailing factors in HD were pre-planned by distance and how they felt during performance [38% (*N* = 8); Figure 4A], and pre-planned by distance and pitstops [38% (*N* = 8); Figure 4A], whereas the prevailing factors in HH were split; pre-planned by distance and how they felt during performance [43% (*N* = 9); Figure 4B], and pre-planned by distance and pitstops [33% (*N* = 7); Figure 4B].

The timing of application of per-cooling strategies were not different in HD and HH by the 60% (*N* = 21) that reported using different strategies in both conditions (Figure 4). The interview findings showed that in HD, the competitive athlete based the timing on elapsed distance, how they felt during the race and when pits stop (transition) were available:

“Well, I would drink the cold-water and pour the cold-water when I was on the bike, which I used in distance as my guide, and then, applied more if I felt like I needed more. The cold-towels were in transition, so that was between swim and bike, and bike and run and I would quickly just press it on my face wiping the sweat away, and then, place it on the back of my neck whilst I checked into my bike and running shoes.”

Thereby, in HH, the competitive athletes based the timing of strategy on distance and how they felt during the race only:

“I based the timing off distance that I was covering on the bike, and then, also off how I felt, so, again, if I was feeling uncomfortable, I would drink and pour more over myself.” and “one thing that was different to the Barcelona race in HD conditions was that I used a lot more water in the Hawaii race compared to the Barcelona race.” And “I think I used about 1L more water in Kona because I kept feeling like I wanted to pour more water over myself to make me feel more comfortable.”

In HD, the professional athlete based the timing of their strategies on elapsed distance only:

“I drank periodically on the bike based on the distance that I was covering” and “Well the bike leg of an IRONMAN is 180.25 km, so every 10 km I would have about 2 sips of my cold-water and I had 2 × 2 L bottles on my bike. I would easily get through 1 and half of those before I get on the run.”

In HH, the professional athlete utilised the same timing as in HD conditions, however, they incorporated ingestion and pouring at each interval:

“I drank periodically on the bike based on the distance that I was covering” and “Well the bike leg of an IRONAMN is 180.25 km, so every 10 km I would have about 2 sips of my cold-water, and then, pour some on myself. I would roughly get through 2 × 2 L bottles.”

These findings show that the competitive and professional athlete employed similar or the same timings for cold-water ingestion and/or pouring in both HD and HH conditions however, the quantity of water used in HH conditions was greater (~1L and ~0.5L) compared to HD.

### Justification of Type and Timing of Per-Cooling Strategy(ies)

**Figure 5:**
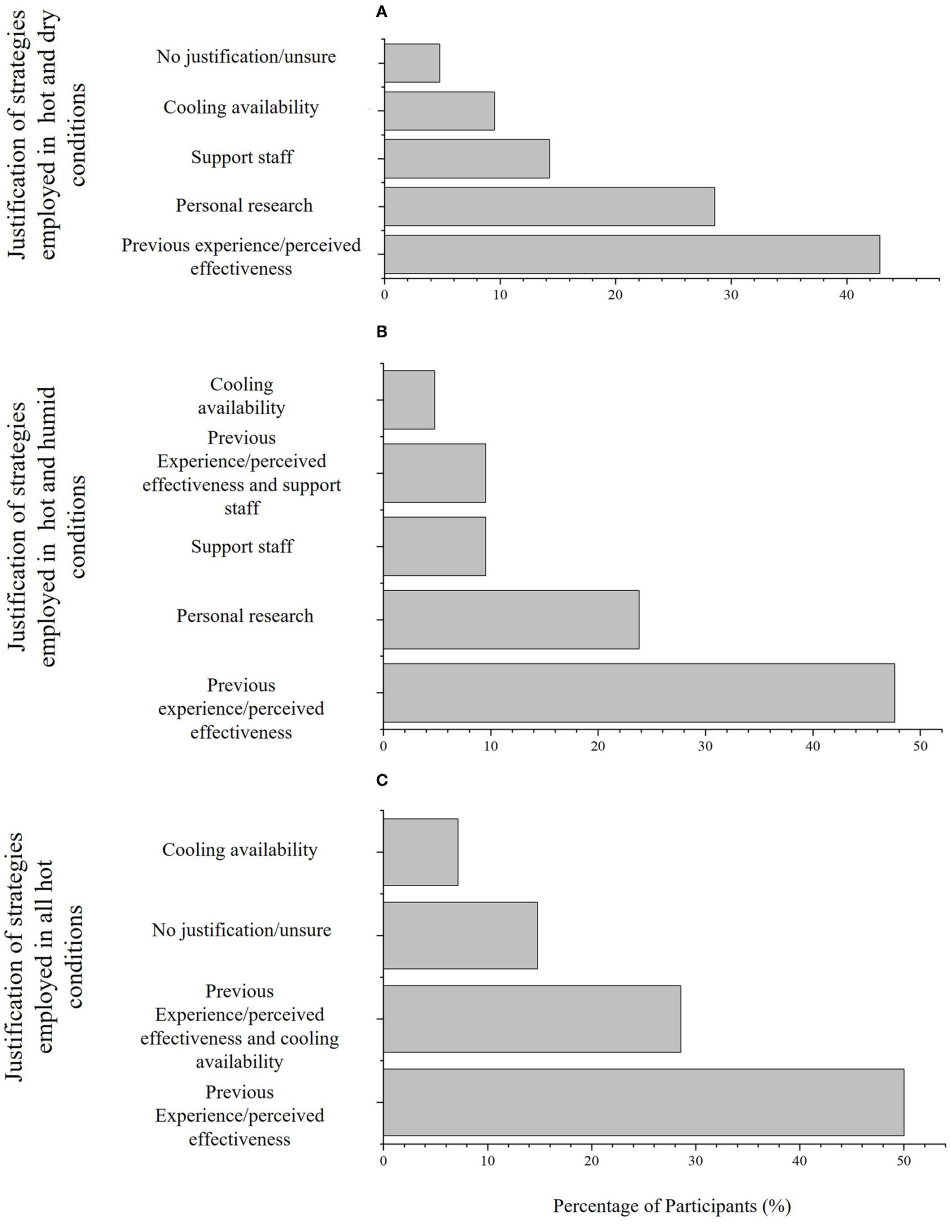
**(A)** How the type and timing of strategies employed in hot and dry conditions by 60% (*N* = 21) participants who use different strategies depending on condition were established, **(B)** how the type and timing of strategies employed in hot and humid conditions by 60% (*N* = 21) participants who use different strategies depending on condition were established, and **(C)** how the type and timing of strategies employed in in all hot conditions by 40% (*N* = 14) participants who employed the same strategy regardless of condition were established.

Participants reported four justifications for type and timing of strategies used in HD (Table 3). The prevailing justification in HD was previous experience/perceived effectiveness (43% of participants; Figure 5A), followed by personal research (29% of participants; Figure 5A). There were five justifications for type and timing of strategies employed in HH (Table 3). Similarly, the prevailing justification in HH was previous experience/perceived effectiveness (48%; Figure 5B), followed by personal research (23% of participants; Figure 5B). There were seven justifications for type of strategies employed in all hot conditions (Table 3). The prevailing justification in all hot conditions was previous experience/perceived effectiveness (50%; Figure 5C).

In HD, the competitive athlete established which strategy to employ and when to apply it based on experience and personal research:

“I typically use the event website together with footage from past races. For example, for IRONMAN Barcelona I watched footage from the year before, and I saw that one of the professional athletes were utilising the transitions to drink and pour cold-water over themselves and had cold towels in coolers. So, I thought I would try that out and see whether it worked for me.”

In HH, the competitive athlete established which strategy to use and when to apply it based experience and personal research:

“Again, I use the event website in combination with footage of past races in the conditions that I am racing in [....] I was watching some footage of the race in Hawaii from the year before and I saw one of the professional athletes using ice slushy ingestion, so I thought it might work for me as well.”

On the other hand, in HD, the professional athletes established which strategies to use and when to apply based on experience and support staff (e.g., sport scientists):

“After competing in the KONA world championships, I wanted to work with my sport scientist again ahead of competing in the IRONMAN OMAN to trial different cooling methods again, such as cold-water ingestion and pouring in the simulated conditions that I would be competing in using an environmental chamber [....] This trial-and-error approach has really helped me figure out which method is not only beneficial but also practical.”

The justification was the same in HH for professional athletes:

“Before competing in the IRONMAN World Championships in KONA, I worked closely with my sport scientist to trial different cooling methods such as cold-water ingestion and pouring in the simulated conditions that I would be competing in using an environmental chamber [....] I found this an effective method to determine which method would best work for me.”

### Perceived Effectiveness

There was no difference between perceived effectiveness of heat mitigation strategies by the 40% (*N* = 14) that employed the same heat mitigation strategies in all hot conditions. One hundred percent (*N* = 14) rated their heat mitigation strategies as 1 (“not effective for minimising performance impairments and heat related illnesses”). There was no difference between perceived effectiveness in HD and HH in the 60% (*N* = 21) of participants that employed different heat mitigation strategies depending on the condition. Fifty-seven percent (*N* = 12) of the 60% (*N* = 21) rated their strategies in HD and HH as 3 (“*Sometimes effective and sometimes not effective”*); whereas, 43% (*N* = 9) of the 60% (*N* = 21) rated their strategies in HD and HH as 4 (“*Effective for minimising performance impairments*”).

The competitive athlete perceived the effectiveness of type and timing of strategies in HD to be 3 (“*Sometimes effective and sometimes not effective”*) which was related to experience:

“I think in terms of performance there were positives and negatives of the strategies that I used. I think the cold-water ingestion and pouring water worked and it really helped me on the bike leg; for example, whenever I did not feel comfortable from the heat I would drink and pour again, which reset me back to feeling comfortable again, so that was a positive.... the cold towels provided an instant benefit, but the benefits did not last very long and made me uncomfortable if I was wearing them for a long period of time [...] On reflection I should have practised this strategy before competing as it was new to me.”

The competitive athlete perceived the effectiveness of type and timing of strategies in HH to be 4 *(“Effective for minimising performance impairments”)*:

“I think the cold-water ingestion and pouring worked well for me […..] I felt a lot better and more comfortable using that strategy after more practise, and that helped with my performance during this race.”

On one hand, the professional athlete thought that the effectiveness of their type and timings of strategies in HD was 4 *(“Effective for minimising performance impairments”)*:

“I felt really comfortable in terms of the conditions when I was there and during the race, I actually felt good, the best that I have felt whilst competing in hot conditions for sure, which I think was reflected in the race outcome.”

The professional athlete thought that the effectiveness of their type and timings of strategies in HD was 4 *(“Effective for minimising performance impairments”)*:

“...with cold-water my performance has continued to improve.”

**Figure 6:**
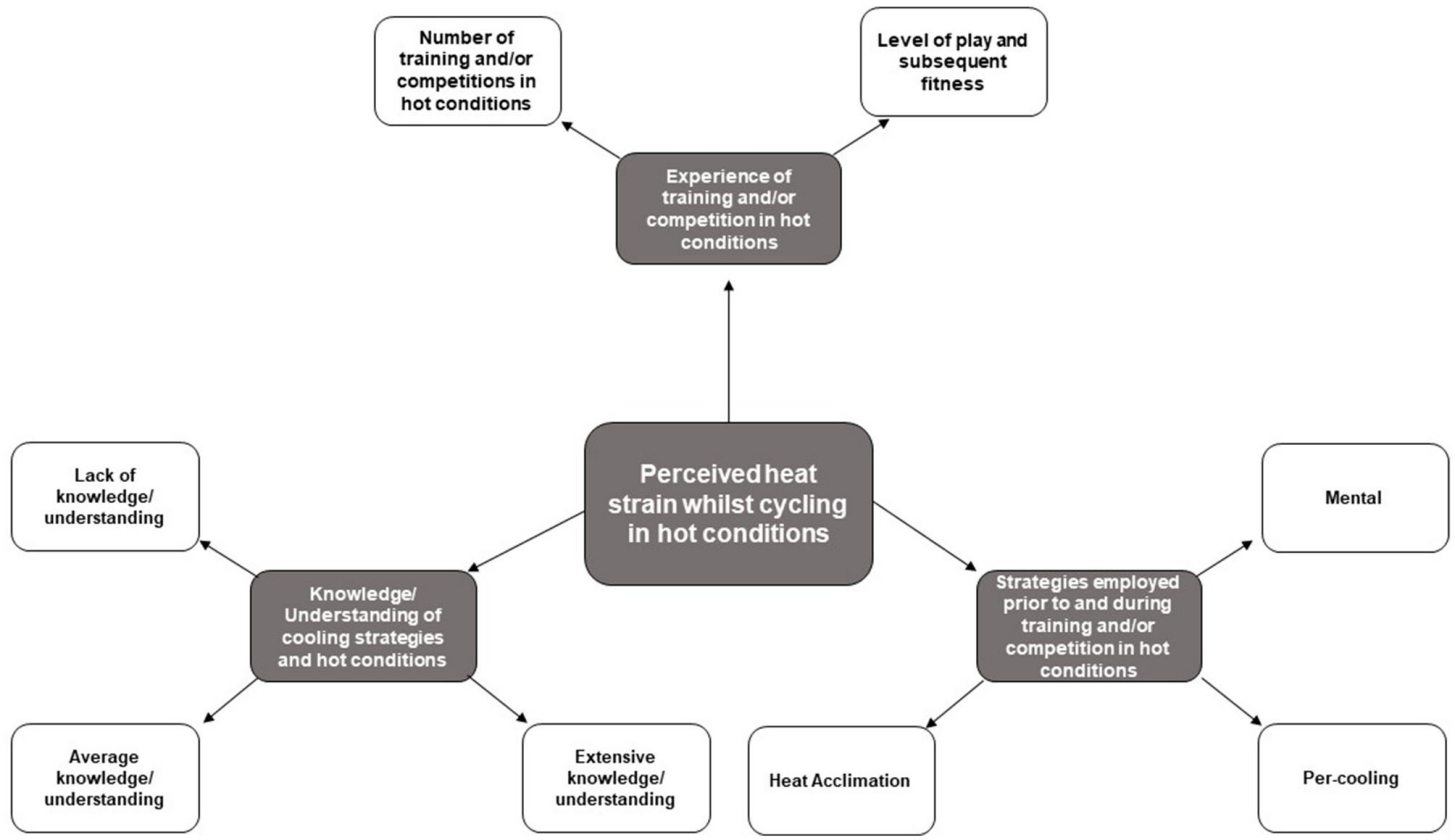
Potential factors influencing perceived heat strain during cycling in hot conditions based on the experiences of 3 cyclists-triathletes. Themes are shaded in grey boxes and subthemes are in white boxes.

Both competitive and professional athlete agree that mental heat mitigation strategies can be effective in both conditions (Figure 6). The competitive athletes found imagery beneficial for minimising heat related illnesses and performance impairments:

“It makes me feel better because I know that when I use that cooling, I will feel more comfortable, and I think knowing what the cooling strategies feel like because I have used them a lot helps with my performance because it gives me something to work towards, i.e., getting to the transition sooner.”

On the other hand, the professional athletes also found positive self-talk and locus of control beneficial for minimising performance impairments:

“I had come to the race to win and that I was going to win, and I did win. So, now when I go into a race, I make sure that I have controlled everything that I can control prior to the race, so that on race day I feel confident that I will win.”

### Comparison of Participant Level

Recreational athletes employ the same type of strategies regardless of environmental condition (*N* = 10). The most reported strategy was cold-water ingestion (*N* = 7). The timing of application was based on when pit stops were available only (*N* = 7). There was no clear justification for strategy type and timing. As a result, recreational athletes' performance was perceived as impaired, and they suffered from heat related illnesses.

Majority of competitive athletes employed different strategies depending on environmental condition (*N* = 11/15). The most reported strategy (*N* = 9) was cold-water ingestion and pouring. The timing of application was pre-planned based on distance, how they felt during and pitstop availability (*N* = 9). Justification of the type and timing of strategies was based on previous experience/perceived effectiveness (*N* = 9). Additional strategies (e.g., cold towels, ice vests) were sometimes added with cold-water ingestion and/or pouring based on perceived effectiveness (*N* = 6). The interview findings revealed that using professional athletes as role models influenced their heat mitigation strategy's type and timing. The perceived effectiveness of these strategies was sometimes effective and sometimes not effective for minimising impairments to performance and heat related illnesses. Therefore, the competitive athletes in this sample have yet to master their heat mitigation strategies for training and/or competing in hot conditions (HD and HH).

All professional athletes employed different strategies depending on environmental condition (*N* = 10). Cold-water ingestion and pouring was the most reported strategy in both HD and HH. Timing of application was pre-planned based on distance and how they felt during (*N* = 10). The justification of strategies used was based on previous experience/perceived effectiveness (*N* = 10). The strategies used were perceived as effective for minimising performance impairments but not heat related illnesses.

## General Discussion

The aim of this study was to investigate the type, timing, and justification of per-cooling strategies employed by athletes (cyclists-triathletes) during training and/or competitions in hot and dry (HD) and hot and humid (HH) conditions.

### Main Findings

1. Cold-water ingestion was the most employed strategy in HD, whereas a combination of cold-water ingestion and pouring was the most employed strategy in HH.
2. Timing of application was pre-planned based on distance in both conditions, supplemented with how participants felt during when pit stops are available in HD, and how participants felt during in HH.
3. The prevailing justifications for type and timing of strategies was previous experience/perceived effectiveness (e.g., trial and error).
4. There was no difference in perceived effectiveness of type and timing of strategies employed in HD and HH.
5. There is a difference in the type, timing, justification, and perceived effectiveness of heat mitigation strategies between recreational, competitive, and professional athletes.
6. Competitive athletes found benefits from mental strategies, such as imagery and modelling; whereas professional athletes found benefits from positive self-talk and locus of control during competition in HD and HH.

The benefits of cold-water ingestion can be summarised as directly cooling core organs and circulating blood, enhanced thermal sensation through thermoreceptors in the mouth and gut, and can be complementary to existing pre-event hydration or nutrition supplementation strategies (e.g., combine with carbohydrates and minerals; James et al., 2015; Bongers et al., 2017). Mechanistically, the thermal stimulus to elicit a phase change from cold to warm water draws heat from internal tissue, reducing temperatures proximal to the gut directly and indirectly cools other regions, as blood of a lower temperature circulates the body. Therefore, unlike external cooling, internal cooling often displays minimal changes in T_sk_, but prompt changes in T_core_/T_rectal_, reflecting the cooling site proximity to core organs, and typical T_core_/T_rectal_ measurements in the gut (e.g., pill) or rectum (e.g., thermistor probe). Cooling *via* the mouth and gut may also positively influence indices of perceived thermal strain (i.e., thermal comfort and sensation), as a consequence of the relative prominence of thermoreceptors in these regions (Villanova et al., 1997; Flouris, 2011). The systemic nature of cooling the periphery through the bloodstream does not lead to the same concerns around localised external cooling, with the maintenance of dexterity and muscle temperature. Therefore, using cold-water ingestion targets a physiological change in T_core_/T_rectal_. In contrast to the strategies employed in the current study, previous findings have highlighted that HD greatly favours evaporation, so cycling in the desert may be an ideal situation for self-dousing with water, as most of it is likely to evaporate (Morris and Jay, 2016). In contrast, a combination of strategies was employed by endurance athletes in Racinais et al. (2021) study showing that 93% of participants employed per-cooling (mainly head/face water dousing/pouring and cold-water ingestion) at IAAF World Athletics Championships in HD. Collectively these findings highlight that there is a lack of consensus between which strategy to use (for example cold-water ingestion only, cold-water pouring only, or a combination of the two) during training and/or competition in HD conditions by endurance athletes.

In contrast to HD, HH conditions make the process of evaporation increasingly difficult, therefore, cold-water pouring may not be the best method whilst cycling in HH (Morris and Jay, 2016). This poses the question as to whether internal cooling *via* cold-water ingestion would be more favourable during HH. Morris et al. (2014) examined local sweating activity, as well as T_core_ and T_sk_, and found that immediately after cold-water ingestion, a sudden drop in local sweat rates occurred at the back, forehead, and forearm. All of which remained depressed for several minutes, despite the fact that T_core_ and T_sk_ were unaltered throughout. Upon further investigation by administering aliquots of water of equal volume and temperature to the mouth *via* swilling or directly into the stomach *via* a nasogastric tube, it was determined that the reductions in sweating were due to signalling from independent thermoreceptors that are probably located in the stomach and/or small intestine without input from thermoreceptors located in the deep body core or skin (Morris et al., 2014). Morris et al. (2016) conducted a subsequent study examining ice slurry ingestion during exercise and found similar results, with sweating drastically reduced following ingestion, without changes in T_core_/T_rectal_ or T_sk_. Perhaps more important than the changes in local sweat rate, Morris et al. (2016) also measured environmental parameters, such as air velocity, ambient temperature and humidity, and designed the experiment in order to estimate heat loss from all avenues of heat transfer. Critically, alterations in evaporative potential due to differences in sweating were determined (Bain et al., 2012). The findings showed that compared to a 37°C drink, the reduction in evaporative heat loss with 1.5 and 10°C fluid ingestion was approximately equal to the additional internal heat transfer obtained with these drinks. Even more surprising was that in the follow up study during which participants ingested ice slurry drinks, the reduction in sweating compared to a 37°C drink was so great that it exceeded the internal heat transfer to the ice slurry, despite the extra internal heat loss due to the latent heat fusion. As such, ice slurry ingestion led to a greater, not smaller, net heat storage compared to a 37°C drink. It must be acknowledged though that a distinct advantage of ingesting cold fluids is that all internal heat transfer is 100% efficient, whereas reductions in sweating in an HH may not mean that evaporation will be equally impacted. Sweat must evaporate to provide a cooling effect, and if it is simply sweat that will ultimately drip off the body anyway that is reduced then cold-water ingestion will likely confer an advantage. Collectively, these mechanistic findings suggest that the cyclists-triathletes in the current study are using the incorrect per-cooling strategies for the condition that they are training and/or competing in. Specifically, cold-water pouring should be employed in HD and cold-water ingestion should be employed in HH. The impact of these strategies on cycling performance have yet to be investigated.

The competitive and professional athletes reported using mental strategies, such as imagery and modelling, and positive self-talk (PST) and locus of control (LOC), respectively, when preparing for competitions in hot conditions (Figure 6). These strategies helped to cope with the discomfort experienced during cycling in HD and HH by creating a cold feeling. This concept was explored in Coudevylle et al. (2019) review, in which the benefits of mental techniques (such as hypnosis) in relation to heat or heat exposure were discussed. For example, Jussiau et al. (2002) showed an increase in heat detection and heat-pain thresholds after a hypnosis intervention. In addition, Younus et al. (2003) showed that the frequency, duration, and severity of the hot flashes were significantly reduced after hypnosis (4 × 1-h/week). These studies demonstrate that mental strategies can be applied and provide benefits in relation to thermoregulation. Future research should aim to investigate whether this also works for cold interventions and whether there were any specific effects on psychological markers (i.e. TC, TS, AF) and the motivation to perform exercise in hot conditions (HD and HH) compared to a thermoneutral/control condition (Coudevylle et al., 2019). The mental strategies employed by professional cyclists-triathletes are supported by previously work demonstrating that PST improved running performance in HD by 8% (Barwood et al., 2008). Therefore, it could be hypothesised that this mental strategy may prove beneficial for other endurance sports such as cycling, and other hot conditions such as HH, however, this has yet to be investigated. In addition to PST, the professional athlete reported controlling as many factors as possible to increase self-confidence going into a competition. This behaviour is classed as locus of control - the extent to which people see the environment as controllable (Schippers and Van Lange, 2006). It has been argued that people who see the environment as controllable feel less tension and are more self-confidence before a competition (Schippers and Van Lange, 2006). Phillips and Hopkins (2020) highlighted that self-confidence had a positive effect on cycling performance. These findings suggest that improving locus of control to view the environment as controllable may improve self-confidence and prove beneficial for cycling performance in hot conditions. Therefore, the current study contributes to the early findings in this research area, demonstrating the potential benefit of using mental strategies/psychological skills training (for example imagery, modelling, PST, LOC) during cycling in hot conditions.

In addition to the type of strategy employed, the current study also highlighted the timing of application in HD and HH. Previous research in the area that is conducted in a laboratory setting commonly provides cold-water *ad-libitum* (Dugas et al., 2009). For example, provided *ad libitum* cold-water (10°C) during a 40-km cycling TT in HH conditions to investigate the frequency and quantity of water consumed. The findings highlighted that cold water was consumed on completion of every 2 kms. The current study supported these findings, highlighting that cold water was ingested based on elapsed distance and how athletes felt in HH (42.85%, *N* = 9; Figure 4). Athletes felt in HH (42.85%, *N* = 9; Figure 4). Athletes also consumed cold water based on elapsed distance and how they felt in HD, with the addition of ingestion at pit stops during competition (38.09%; Figure 4). However, in the athletes that employed the same strategies regardless of competition, cold water ingestion was completed at pit stops only (50%, *N* = 7; Figure 4). Therefore, the optimal timing of cold-water ingestion for thermoregulatory and performance benefits during a self-paced cycling TT in HD and HH are still unknown.

Cyclists-triathletes rely on previous experience/perceived effectiveness when selecting type and timing of strategies. Mechanistically the strategies selected were not the most effective for the desired conditions, however, the effectiveness of these strategies on minimising performance impairments and heat related illnesses have yet to be investigated in these conditions. There was no difference in perceived effectiveness of strategies employed in HD and HH by the 60% (*N* = 21) of participants that reported using different strategies depending on condition. However, the interview findings revealed that there was a difference between perceived effectiveness of strategies between HD and HH for competitive and professional cyclists-triathletes. The professional cyclists-triathletes stated using a trial-and-error approach with heat mitigation strategies which were advised by a sport scientist. The competitive and recreational cyclists had less experience and support compared to the professional cyclists-triathletes, which may explain why 80% of recreational and 60% of competitive cyclists-triathletes employed the same heat mitigation strategy regardless of the condition. Surprisingly, the competitive cyclists-triathletes chose to use the same heat mitigation strategy in both conditions despite their understanding of perceived heat stress (Figure 2). This highlights that there may be a lack of understanding among competitive cyclists-triathletes in what heat mitigation strategies to use (e.g., type and timing) in different conditions (e.g., HD vs. HH) (Figure 6). The interview findings highlighted that the competitive athletes use modelling of professional athletes' behaviour to obtain information on which heat mitigation strategies to use. It is common for fans of elite/professional athletes to mimic behavioural cues (Lynch et al., 2014) or use copying as an effective skill development technique (Abraham and Collins, 2011). However, the use of modelling to determine heat mitigation strategies was a novel finding of the current study.

In addition, the recreational cyclists-triathletes' strategies were perceived as not effective for minimising performance impairments and heat related illnesses. Shendell et al. (2010) identified adult recreational endurance athletes, and in particular less experienced (e.g., first time) participants, as a susceptible and vulnerable population subgroup to heat related illnesses (Shendell et al., 2010). These findings were related to a lack of knowledge in exercising in hot conditions mostly from lack of experience and education on heat related illnesses (Shendell et al., 2010). An additional factor that contributes to this is that recreational athletes train less and at a lower intensity than competitive and professional cyclists-triathletes, implying that they would have a lower physical fitness and/or higher BMI which are risk factors for heat exhaustion (Winkenwerder and Sawka, 2007). The findings of the current study support Shendell et al. (2010) study demonstrating that recreational cyclists-triathletes are at a higher risk compared to competitive and professional athletes due to knowledge/understanding and experience (Figure 6). Therefore, it is important that recreational and competitive athletes are educated on the impairment effect of HD and HH on performance and risk of heat related illnesses together with strategies on how to compete this impairment effect. This education should also cover the risk of modelling wrong heat mitigation strategies in different environmental conditions.

## Limitations and Future Research Recommendations

Despite being derived from a relatively small sample size (*N* = 35), the findings of this study highlight the existing strategies employed by cyclists-triathletes during training and/or competitions in hot and dry and hot and humid conditions. The importance of this is that different types of strategies are employed depending on the condition however the effectiveness of these strategies in these conditions have yet to be explored. Therefore, future larger scale studies should explore the effectiveness of these heat mitigation strategies in hot and dry and hot and humid conditions. The study of Racinais et al. (2021) had an effective approach to pre-race questionnaires. This would also allow for exact conditions on competition day to be recorded and related to questionnaire findings.

It was also reported that cyclists-triathletes do not only use physical heat mitigation strategies, but also mental strategies when preparing for training and/or competitions in hot conditions. Therefore, future research should investigate the mental strategies employed and their effectiveness on minimising performance impairments and heat related illnesses.

As noted in the methodology, questions related to quantity and magnitude of cooling employed were removed from the questionnaire/interview to focus on type, timing, justification, and perceived effectiveness in hot and dry and hot and humid conditions. Reported the quantity of water consumed during a cycling time-trial, which increased over time [0–8 km (~50 ml), 8–16 km (~100 ml), 16–24 km (~260 ml), 24–32 km (~230 ml), and 32–40km (~180ml)] equating to a total consumption of 1.1 ± 0.4 L. This strategy contributed to a completion time of 93 + 3.5 min, however, few studies have investigated the impact of cold-water ingestion on performance and, therefore, it is unclear whether this method was effective at improving TT performance in HH conditions. In contrast to self-paced protocols, exercise to exhaustion protocols often base consumption of cold-water (4°C) on time, with every 15mins being the most reported strategy in both hot and dry (Mündel et al., 2006; Naito and Ogaki, 2017) and hot and humid (Lee et al., 2008). Therefore, the optimal quantity of cold-water to ingest and/or pour in hot and dry and hot and humid conditions is unknown and should be an area for future research to explore.

Finally, the results suggest the need to educate competitive and recreational athletes on heat strain and heat mitigation strategies. Based on the competitive athletes' use of modelling to obtain information on heat mitigation strategies, it may suggest that the best approach to educate competitive and recreational athletes is through role-models (for example professional athletes). This could be conducted through professional athletes together with their support staff giving talks/webinars, which reflect on current and past practise.

## Conclusion

Using mixed-method methodology and a population of cyclists-triathletes, this study identified that cold-water ingestion was the prevalent cooling strategy employed in hot and dry, whereas a combination of cold-water ingestion and pouring was the most reported strategy in hot and humid. In HD, the timing of application was based on elapsed distance, how they felt during and when pitstops were available, compared to elapsed distance and how they felt during in HH. The type and timing of strategies were based on previous experience and perceived effectiveness. There was no difference in the perceived effectiveness of strategies employed in HD and HH. However, the type and timing of strategies have yet to be investigated in the favoured conditions for their effect on minimising performance impairments and heat related illnesses. Mental strategies seem to be promising methods that require further investigation in hot and dry and hot and humid conditions.

Future research should investigate the effectiveness of cold-water ingestion and pouring on performance in hot and dry and hot and humid conditions to determine the optimal type of strategy for cyclists-triathletes to use during training and/or competition in these conditions. The impact of mental strategies should also be investigated further in both isolation and combination with cold-water ingestion, pouring and combined ingestion, and pouring.

## Data Availability Statement

The raw data supporting the conclusions of this article will be made available by the authors, without undue reservation.

## Ethics Statement

The studies involving human participants were reviewed and approved by London South Bank University. The patients/participants provided their written informed consent to participate in this study.

## Author Contributions

FB: lead author, study writing, data collection, and analysis. NG, SR, KM, and SH: conceptualisation, funding acquisition, supervision, and writing-review and editing. All authors have read, approved the final version of the manuscript and made a significant contribution to this study.

## Funding

FB was supported by scholarship from London South Bank University and BodyCap. None of the authors have professional relationships with companies or manufacturers who will benefit from the results of this study. None of the results presented in this study constitutes an endorsement by the American College of Sports Medicine.

## Conflict of Interest

The authors declare that the research was conducted in the absence of any commercial or financial relationships that could be construed as a potential conflict of interest.

## Publisher's Note

All claims expressed in this article are solely those of the authors and do not necessarily represent those of their affiliated organizations, or those of the publisher, the editors and the reviewers. Any product that may be evaluated in this article, or claim that may be made by its manufacturer, is not guaranteed or endorsed by the publisher.
